# *Polygonum multiflorum* extract support hair growth by elongating anagen phase and abrogating the effect of androgen in cultured human dermal papilla cells

**DOI:** 10.1186/s12906-020-02940-5

**Published:** 2020-05-12

**Authors:** Jae Young Shin, Yun-Ho Choi, Jaeyoon Kim, Se Young Park, You Jin Nam, So Young Lee, Jeong Hoon Jeon, Mu Hyun Jin, Sanghwa Lee

**Affiliations:** 1 Research Park, LG Household & Healthcare Ltd, 70, Magokjoongang 10-ro, Gangseo-gu, Seoul, 07795 South Korea; 2 Department of biotechnology, CHA University, 335, Pangyo-ro, Bundang-gu, Seongnam-si, Gyeonggi-do 13488 South Korea

**Keywords:** Dermal papilla, 3D DPC spheroid, Growth factor, Anagen elongation, Dkk1, Dihydrotestosterone, Androgen receptor, *Polygonum multiflorum*

## Abstract

**Background:**

Dermal papilla cells (DPCs) play a key role in hair growth among the various cell types in hair follicles. Especially, DPCs determine the fate of hair follicle such as anagen to telogen transition and play a pivotal role in androgenic alopecia (AGA). This study was performed to elucidate the hair growth promoting effects of *Polygonum multiflorum* extract (PM extract) in cultured human DPCs and its underlying mechanisms.

**Methods:**

The effects of PM extract on cultured DPCs were investigated. Cell viability and mitochondrial activity were measured by CCK-8 and JC-1 analysis, respectively. Western blotting, dot blotting, ELISA analysis, immunocytochemistry and real-time PCR analysis were also performed to elucidate the changes in protein and mRNA levels induced by PM extract. 3D cultured DPC spheroids were constructed for mimicking the in vivo DPs. The hair growth stimulatory effect of PM extract was evaluated using human hair follicle organ culture model.

**Results:**

PM extract increased the viability and mitochondrial activity in cultured human DPCs in a dose dependent manner. The expression of Bcl2, an anti-apoptotic protein expressed dominantly in anagen was significantly increased and that of BAD, a pro-apoptotic protein expressed in early catagen was decreased by PM extract in cultured DPCs and/or 3D DPC spheroid culture. PM extract also decreased the expression of catagen inducing protein, Dkk-1. Growth factors including IGFBP2, PDGF and VEGF were increased by PM extract, revealed by dot blot protein analysis. We also have found that PM extract could reverse the androgenic effects of dihydrotestosterone (DHT), the most potent androgen. Finally, PM extract prolonged the anagen of human hair follicles by inhibiting catagen entry in human hair follicle organ culture model.

**Conclusion:**

Our data strongly suggest that PM extract could promote hair growth by elongating the anagen and/or delaying the catagen induction of hair follicles through activation of DPCs.

## Background

Hair follicles undergo three stages of the hair growth cycle called anagen, catagen, and telogen, which corresponds to the growing, regressing, and resting phases, respectively. Each stage of the hair cycle is classified according to the morphological, functional, and compositional characteristics of hair follicles. Among various cell types that organize the hair follicle, dermal papilla cells (DPCs) play key roles in proliferation and differentiation of hair follicles and controlling hair cycle in each phase [1]. Especially, growth factors, including insulin-like growth factor-1 (IGF-1), hepatocyte growth factor (HGF), vascular endothelial growth factor (VEGF) and keratinocyte growth factor (KGF), from the follicular dermal papilla (DP) stimulate keratinocytes to proliferate and differentiate into the hair shaft during anagen [2, 3]. Transition of hair cycles is also controlled by DPCs. Secreted proteins such as transforming growth factors (TGF) β1, TGF β2, and dickkopf1 (DKK-1) are known to induce transition from anagen to telogen [4]. When telogen is induced, apoptosis and follicular regression occur throughout the hair follicle [5]. During anagen or at the end of telogen, Bcl2 is highly expressed in DPCs, compared to other types of hair follicle cells [6]. Among Bcl2 family proteins, Bcl2 is regarded as one of the most important anti-apoptotic agents. On the other hand, BAD protein, another member of bcl2 family, is known to induce apoptosis [7].

Androgens, steroid hormones that regulate the development and maintenance of male characteristics in vertebrates, also indirectly control hair growth by affecting the activities of the DP [8]. Dihydrotestosterone (DHT) is derived from testosterone by the action of 5α-reductase and is considered to be more potent in triggering hair loss. DHT causes the miniaturization of DP that leads to hair shaft thinning and stimulate the expression of TGF β1 in DP resulting in growth inhibition of epithelial cells [9]. Consequently, prolonged exposure of DHT on DP causes androgenic alopecia (AGA) or hair shaft weakening [10, 11].

*Polygonum multiflorum* (PM) is a species of flowering plant in the buckwheat family *polygonaceae*. It is one of the most popular perennial traditional Chinese medicines called *He Shou Wu* in China and East Asia. PM has long been used as a component for anti-hair loss and anti-hair greying treatment prescriptions [12]. Several reports demonstrated hair growth effects of PM extract. Histological analysis of C57BL/6 mouse cases showed that PM extract increased the size and the number of hair follicles via upregulating β-catenin and sonic hedgehog expressions by both topical and oral applications [13, 14]. Also, anti-androgenic effects of PM extract were reported in several studies with prostate cancer cells, by inhibition of 5-α reductase, a key enzyme for DHT production [15]. Especially, 2,3,5,4′-Tetrahydroxystilbene-2-O-β-D-glucoside (TSG) and emodin, single compounds identified in PM extract, were reported to show hair growth properties. TSG exerted anti-apoptotic effect in C57BL/6 murine follicles [16] and pharmacological effects on age related diseases, resulting in cardio-protective, neuro-protective and anti-hair loss [17]. Concretely, TSG acts as a protector of dopaminergic neurons by regulating Akt, GSK3β and Bcl2/BAD expressions as well as a hypotensive agent in vascular endothelial cells like minoxidil [18, 19]. Emodin was reported to strongly inhibit 5-α reductase activity in benign prostatic hyperplasia [20] and promote topical hair growth in C57BL/6 [21]. Although hair growth stimulating effects of PM extract were reported in several studies using mouse models, detailed biological mechanism for anti-hair loss effects of PM extract has not been elucidated in the human system, especially focused on DPCs.

In this study, we investigated the hair growth promoting effects of PM extract in cultured human DPCs and the underlying molecular and cellular mechanisms. It was found that treatment of PM extract stimulated proliferation and mitochondrial activity in cultured human DPCs. PM extract increased the expression of BCl2, an anti-apoptotic protein and decreased the expression of BAD, a pro-apoptotic protein in cultured DPCs and/or 3D DPC spheroid culture. Also, PM extract decreased the expression of catagen inducing protein, Dkk-1. In addition, the expression of growth factors like PDGF-aa and VEGF, known to be crucial for hair growth, was increased by PM extract treatment. These results clearly demonstrate the potential role of PM extract in promoting hair growth by elongating anagen and/or delaying catagen entry. PM extract was found to prolong the anagen of human hair follicles by inhibiting catagen entry in human hair follicle organ culture model. We observed anti- androgenic effects of PM extract, different from previously reported mechanism related to inhibition of 5α-reductase. It was revealed that PM extract significantly reduced the expression of androgen receptor (AR) induced by DHT and recovered the reduced size of DPC spheroid by DHT treatment which mimicked the hair follicle miniaturization observed in AGA.

In conclusion, our data strongly suggest that PM extract could support hair growth by extending anagen duration and delaying catagen progression and could possibly prevent hair loss by abrogating the effects of androgen which result in hair follicle DP miniaturization, suspected to be a main cause of AGA.

## Methods

### *Polygonum multiflorum* (PM) extract preparation

The dried roots of *Polygonum multiflorum* Thunberg were purchased from Humanherb (product no. G152150411, Daegu, Korea) in August 2016 and identified by Prof. Seok-Seon Roh in the College of Korean Medicine, Daejeon University. The voucher specimen was stored in LG households and healthcare Natural Plant Center (LG008462). The dried roots of PM (40 g) were extracted with 50% aqueous ethanol for 2 days at room temperature, and then filtered through Whatman No. 4 filter paper. The filtrate was concentrated by rotary evaporator under reduced pressure to give 50% aqueous ethanol extract (11.69 g, 29% yields).

### Cell culture

Human Dermal Papilla cells (DPCs) were purchased from Promocell (Promocell, Heidelberg, Germany). DPCs were cultured in a basal medium supplemented with Supplement Mix which contains 4% fetal calf serum, 0.4% bovine pituitary extract, 1 ng/ml basic fibroblast growth factor, and 5 μg/ml insulin. Cells were maintained in humidified incubator at 37 °C with 5% CO_2_.

### Cell viability assay

The effects of PM extract on DPCs cell viability were examined using CCK-8 assay (Dojindo, MA, USA) and JC-1 mitochondrial membrane potential assay (Abcam, Cambridge, UK) following the manufacturer’s protocols. Briefly, DPCs (3 × 10^3^ cells/well) were seeded in 96-well plates and cultured for 24 h. Triplicate cultures of DPCs were treated with various concentrations of PM extract and cultured for another 24 h. The NADH and NADPH generation was determined by CCK-8 assay which indicates the cell viability. The absorbance at 450 nm was measured using Epoch micro plate spectrophotometer (BioTek, VT, USA). The mitochondrial membrane potential was measured by JC-1 staining. DPCs were stained with 1 μM JC-1 solution. JC-1 aggregate form was measured at 590 nm for emission (535 nm for excitation) and monomer form at 530 nm for emission (475 nm for excitation). Distribution of JC-1 aggregate and monomer was detected by immunofluorescence microscopy using EVOS™ FL Auto2 Imaging System (Thermofisher scientific, MA, USA).

### 3D DP spheroid construction & cryo section

For assessing the size of DPC spheroids, DPCs (2x10^5^cells/well) were seeded in 24well Hydrocell plates (Nunc, Roskilde, Denmark), which have chemical coated surface for preventing cell attachment, consequently promoting spheroid formation. For immunocytochemical analysis, DPCs (1.5x10^4^cells/well) were seeded in 96well u-bottom hydrocell plate (Nunc, Roskilde, Denmark). Cells were incubated for 72 h to generate spheroids with / without PM extract. Then the size of spheroids was measured by microscopic photography (Leica, Wetzlar, Germany) and sorted by diameter.

For immunocytochemistry, 3D DP spheroids were embedded in OCT compound for cryo section. Then OCT compound embedded 3D DP spheroids were frozen at − 80 °C for more than 1 h. The frozen 3D DP spheroids were sliced in 15 μm thick using pre-chilled Thermo Shandon Cryotome (Thermofisher scientific, MA, USA).

### DKK-1 ELISA

DPCs (2x10^5^cells/well) were seeded in 24 well plates and cultured for 24 h. Cells were treated with various concentrations of PM extract for 24 h. Then, culture supernatants were collected and the amounts of DKK-1 were measured using HUMAN DKK-1 DuoSet ELISA (R&D systems) kit, according to the manufacturer’s instruction. Briefly, anti-human Dkk-1 capture antibody was plated to 96well plate for 12 h at room temperature. Wells were blocked with blocking buffer (1% BSA in PBS, pH 7.2–7.4), and 100 μl of supernatant samples were added to wells in triplicate and incubated for 2 h at room temperature. After anti-human DKK-1 detection antibody was treated for 12 h at 4 °C, 100 μl of the working dilution of Streptavidin-HRP was added to each well and incubated for 20 min. Wash steps were included between each step. After incubation with 100 μl of substrate solution for 20 min, absorbance at 450 nm was measured using microplate reader. Background wavelength correction was performed at 540 nm.

### Bcl2, androgen receptor (AR) immunocytochemistry

3D cultured DP spheroids (three pre-made spheroids/well) were seeded in 96well plates and cultured overnight. After PBS wash, DP spheroids were fixed with 4% paraformaldehyde at room temperature for 10 min. Cells were then permeabilized with PBS containing 0.1% triton x-100 and blocked with PBS containing 5% FBS and 1% BSA. After consecutive incubation with primary antibodies (200:1 dilution, Abcam, Cambridge, UK) at 4 °C for 12 h and alexa 488 nm or alexa 594 nm conjugated secondary antibodies (1000:1 dilution, Thermofisher scientific, MA, USA) at room temperature for 1 h, nucleus were stained with DAPI (2000:1 dilution, Thermofisher scientific, MA, USA) in the dark for 10 min. High resolution fluorescence images were taken using EVOS™ FL Auto2 Imaging System (Thermofisher scientific, MA, USA).

### Dot blot array of growth factors

Human growth factor antibody array membrane kit was used (Abcam, Cambridge, UK) to evaluate the changes in growth factor profiles of DPCs by PM extract treatment. Total of 41types of human growth factors could be analyzed at once. Briefly, DPCs (1x10^5^cells/well) were seeded in 24well plates and cultured overnight. Cells were treated with 20 μg/ml of PM extract for 24 h, and then culture supernatants were collected for growth factor analysis. Fresh medium and culture supernatant from non-treated cells were used as blank and control, respectively. Conventional immunoblot process was performed following the manufacturer’s guide. Biotin-conjugated anti-cytokines antibodies were used as primary antibody and HRP-conjugated streptavidin was used for chemiluminescence detection. The resulting blots were analyzed under identical condition using chemiluminescence detector Fusion FX5 (Vilber Lourmat, France).

### Western blot

DPCs (1x10^6^cells/dish) were seeded in 100 mm culture dishes and cultured for 24 h. PM extract were treated at concentrations of 10 and 100 μg/ml for 24 h. Cells were then washed with ice-cold PBS and lysed on ice in M-PER buffer (Thermofisher scientific, MA, USA) supplemented with Complete™ protease inhibitor cocktail and phosphatase inhibitor (Roche, Indianapolis, IN, USA).

40 μg of protein was analyzed by western blotting with appropriate antibodies to evaluate protein expression; DKK-1 (1000:1 dilution, Cambridge, MA, USA), AR (1000:1 dilution, Santa Cruz, CA, USA), Erk (p44/42) (1000:1 dilution, Santa Cruz, CA, USA), GAPDH (2000:1 dilution, Santa Cruz, CA, USA). Western blot was analyzed by chemiluminescence detector (Vilber Lourmat, France).

### mRNA analysis

DPCs (1x10^6^cells/well) were seeded in 6 well plates and cultured for 24 h. Then PM extract was treated at concentrations of 20 μg/ml and 50 μg/ml for 24 h. Total RNA was isolated using RNA isolation kit (Qiagen, RNeasy mini kit) according to the manufacturer’s guide. After RNA isolation, cDNA was synthesized by reverse transcription using eCube cDNA synthesis kit (philekorea, Korea) with PCR thermocycler (R&D systems, MN, USA), according to the manufacturer’s protocol. cDNA obtained from control cells and PM extract treated cells were subject to real-time PCR analysis. TaqMan probes used in this study were as follows: GAPDH assay id 4352934E; BAD assay id Hs00188930_m1; Bcl2 assay id Hs00608023_m1. TaqMan One-Step RT-PCR Master Mix Reagents (Life Technologies, CA, USA) was used. The PCR reactions were performed on ABI7500 Real Time PCR system following the manufacturer’s protocol. The resulting data were analyzed with ABI software.

### Human hair follicle organ culture and hair cycle scoring

Human scalp skin specimens were obtained from patients undergoing reconstructive plastic surgery after obtaining informed consent, following Declaration of Helsinki principles. The study was approved by the Institutional Review Board of the CHA Bundang Medical Center (IRB No. 2018–09-009).

Anagen human hair follicles were isolated by micro-dissection and maintained in William’s E medium (WelGENE, Kyungsan, Korea) supplemented with 10 μg/ml insulin (Sigma-Aldrich, MO, USA), 10 ng/ml hydrocortisone (Sigma-Aldrich, MO, USA), 20 mM HEPES (Invitrogen-Gibco-BRL, NY, USA), and 1x antibiotic-antimycotic (Invitrogen-Gibco-BRL, NY, USA) for 1 day.

Each group of 20 isolated hHFs was cultured in medium containing PM extract at concentrations of 2, 20 and 50 μg/ml. On every third days, medium was replaced and hHFs were photo-documented. Hair cycle stages of cultured human hair follicles were determined on day 0 and 6, according to hair cycle guideline [22, 23].

### Statistical analysis

All experimental data were presented as the mean ± standard deviation (S.D.) of at least three independent experiments. Experimental results were analyzed using the SigmaPlot (Systat Software Inc., IL, USA). The statistical significance of the difference was determined using Student’s t-test. The value of *p* < 0.05 was considered statistically significant.

## Results

### PM extract increased cell viability and mitochondrial activity of cultured human DPCs

**Fig. 1:**
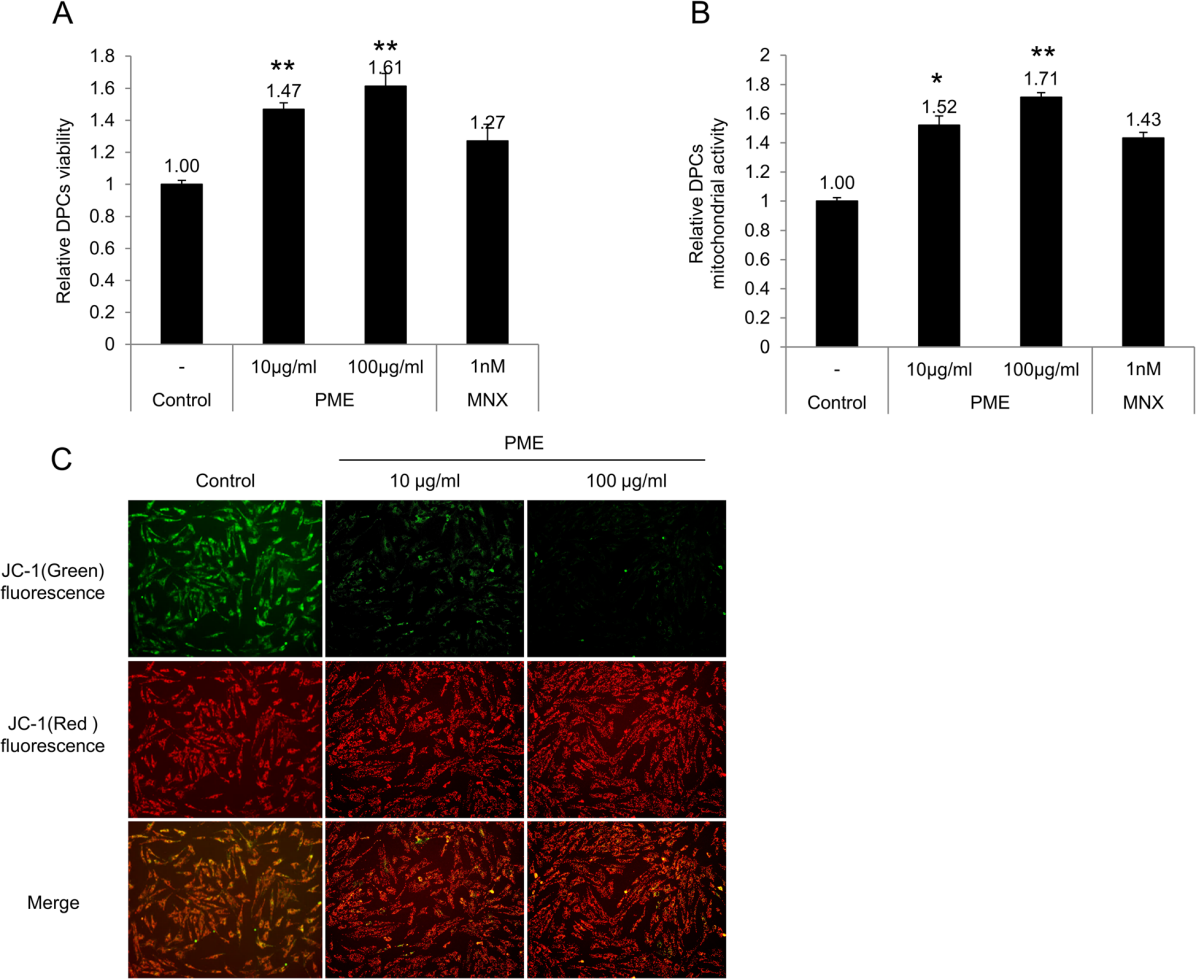
Effect of PM extract on DPCs’ viability and mitochondrial activity. DPCs were treated with 10 μg/ml and 100 μg/ml of PM extract for 24 h. **a** Cell viability of DPCs treated with PM extract was measured using CCK8 assay. **b** JC-1 aggregates (A_590_)/monomer (A_530_) ratio of DPCs treated with PM extract. **c** Distribution of JC-1 aggregates (590 nm, red) and monomer (530 nm, green) by immunofluorescence photography. Data are presented as mean ± SD. *n* > 3 for each group. *p < 0.05, **p < 0.01 compared with control group

The effects of PM extract on cell viability and mitochondrial activity in cultured human DPCs were investigated using CCK-8 assay and JC-1 mitochondrial staining, respectively. CCK-8 assay represents viability of cells via measuring dehydrogenase activity. JC-1 staining labels mitochondria according to membrane potential, high with red, low with green. As shown in Fig. 1a, treatment of PM extract increased cell viability in a dose dependent manner, resulting in 47 and 61% increase at concentrations of 10 μg/ml and 100 μg/ml, respectively. Minoxidil, most well-known topical hair growth stimulating agent, used as a positive control, also increased cell viability by 27% at 1 nM (Fig. 1 a). We hypothesized that increased cell viability might be a consequence of stimulated mitochondrial activity in cultured DPCs.

To verify this hypothesis, the mitochondrial activity of DPCs was evaluated by JC-1 staining, which measures mitochondrial membrane potential. As shown in Fig. 1b, PM extract also increased mitochondrial membrane potential in a dose dependent manner. The mitochondrial membrane potential of cultured human DPCs was increased by 52 and 71% with treatment of PM extract at concentrations of 10 μg/ml and 100 μg/ml, respectively. Minoxidil increased the mitochondrial potential by 43% when treated at 1 nM concentration (Fig. 1b). Highly activated mitochondrial membrane potential could be visualized by fluorescence microscopy where red dots were increased and green dots were decreased by PM extract in cultured human DPCs (Fig. 1c). Our data suggest that PM extract enhanced the viability of DPCs by stimulating the mitochondrial activity, especially mitochondrial membrane potential.

### PM extract decreased the expression of DKK-1 and BAD while increased the expression of Bcl2

**Fig. 2:**
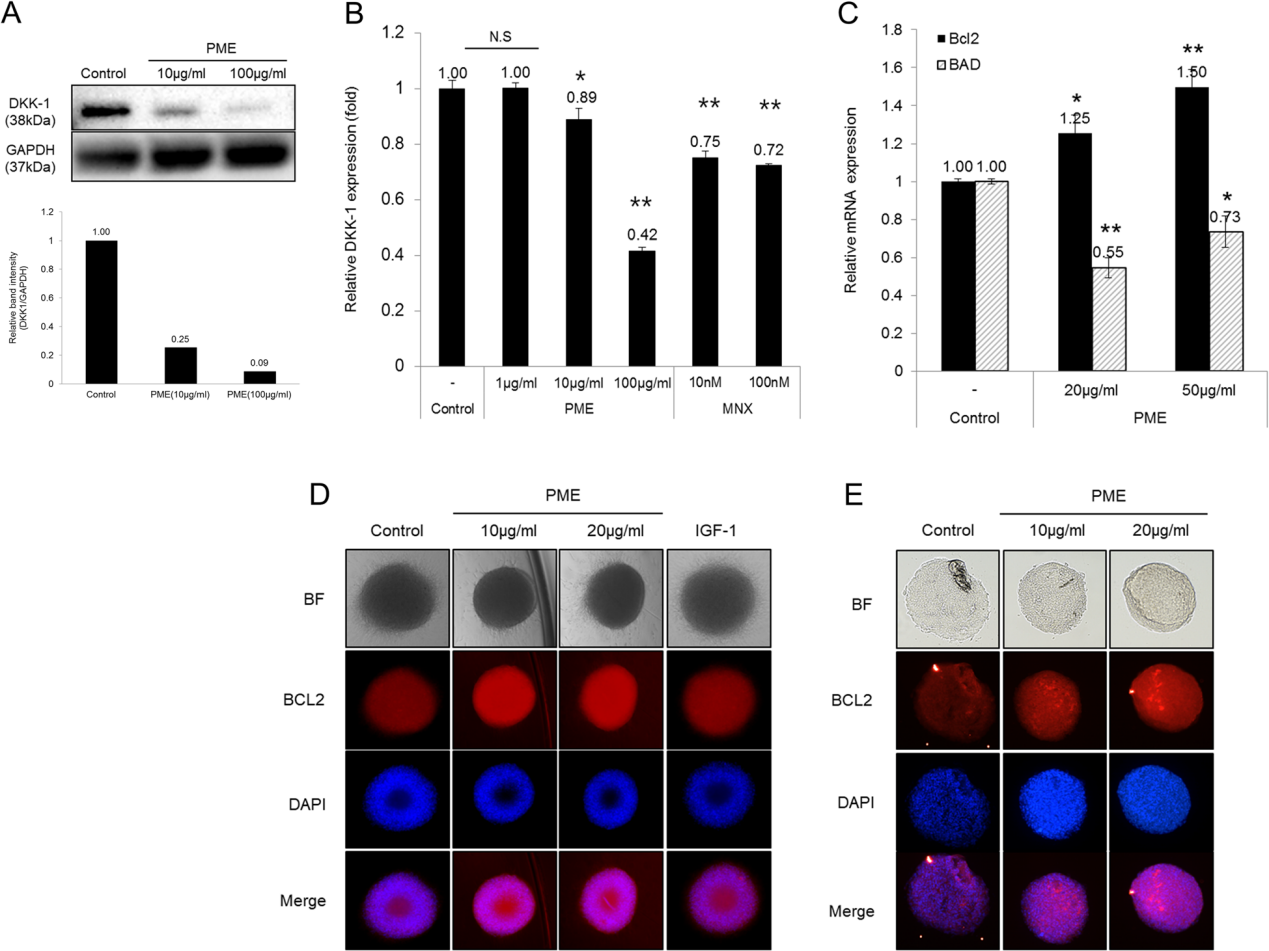
Effect of PM extract on DPCs’ protein expression related to hair cycle. DPCs (1 × 10^6^ cells/100 mm dish) were treated with 1 μg/ml, 10 μg/ml and 100 μg/ml of PM extract for 24 h. For DP spheroids, 10 μg/ml and 20 μg/ml of PM extract were treated at the start of spheroid formation. **a** The protein level of DKK-1 in whole cell lysate of 2D cultured human DPCs and (**b**) supernatant. **c** Relative mRNA expression of Bcl2 and BAD in 2D cultured DPCs. **d** Bcl2 expression in 3D DP spheroids. **e** Bcl2 expression in cryo-sectioned 3D DP spheroids. Data are presented as mean ± SD. *n* > 3 for each group. *p < 0.05, **p < 0.01 compared with control group

Hair follicle enters a regression phase, catagen, in response to various micro-environmental stimuli. DKK-1, one of the most important proteins that induce catagen in hair follicles, is involved in apoptosis in various cell types [24, 25]. When hair follicles enter catagen, hair follicle cells undergo apoptosis, resulting in follicular regression and eventually hair loss [26]. As shown in Fig. 2, DKK-1 protein levels were significantly decreased by PM extract both in whole lysate and culture supernatant of cultured human DPCs in dose dependent manners, revealed by western blot and ELISA analysis, respectively (Fig. 2a, b). Because DKK-1 plays pivotal role in inducing catagen in murine and human hair follicles, it could be conjectured that PM extract could delay catagen entry by down-regulating DKK-1 expression in DPCs.

Different from other cell types in hair follicles which undergo apoptosis during catagen, DPCs are considered to be resistant to proapoptotic environment throughout hair cycles. This anti-apoptotic property is thought to be conferred by highly expressed Bcl2 level in DPCs [27]. Bcl2 family proteins play an important role in promoting cell survival and inhibiting the actions of pro-apoptotic proteins. They also activate DNA repair mechanism to prevent cell death in follicular stem cells [28]. The treatment of PM extract had shown to increase Bcl2 and decrease BAD mRNA expression in cultured human DPCs (Fig. 2c). It was suggested that the Bcl2/BAD ratio could represent a parameter for anti-apoptotic status since BAD induces apoptosis [29]. PM extract at concentrations of 20 μg/ml and 50 μg/ml increased the Bcl2/BAD ratio to 2.27 and 2.05 folds (calculated based on Fig. 2c), suggesting possible anti-apoptotic effects of PM extract in cultured DPCs. To confirm the Bcl2 inductive effect of PM extract in mimicking the DP in hair follicles, 3D spheroid cultures of DPCs were constructed in the presence of PM extract. The expression of Bcl2 protein was assessed by immunocytochemistry in whole and 15 μm thick cryo-sectioned 3D spheroids. As shown in Fig. 2d and e, the expression of Bcl2 protein was significantly increased by PM extract. Treatment of PM extract at concentrations of 10 μg/ml and 20 μg/ml resulted in 67 and 65% increase of Bcl2 expression in whole spheroids, respectively, as measured by fluorescence intensity. The Bcl2 expression in cryo sectioned spheroids was markedly increased by 67 and 105% with 10 μg/ml and 20 μg/ml of PM extract, respectively. Our data suggest that PM extract could prolong the anagenand put off catagen entry by increasing the expression of Bcl2, and by decreasing the expression of DKK-1.

### PM extract increased the expression of growth factors in cultured DPCs

**Fig. 3:**
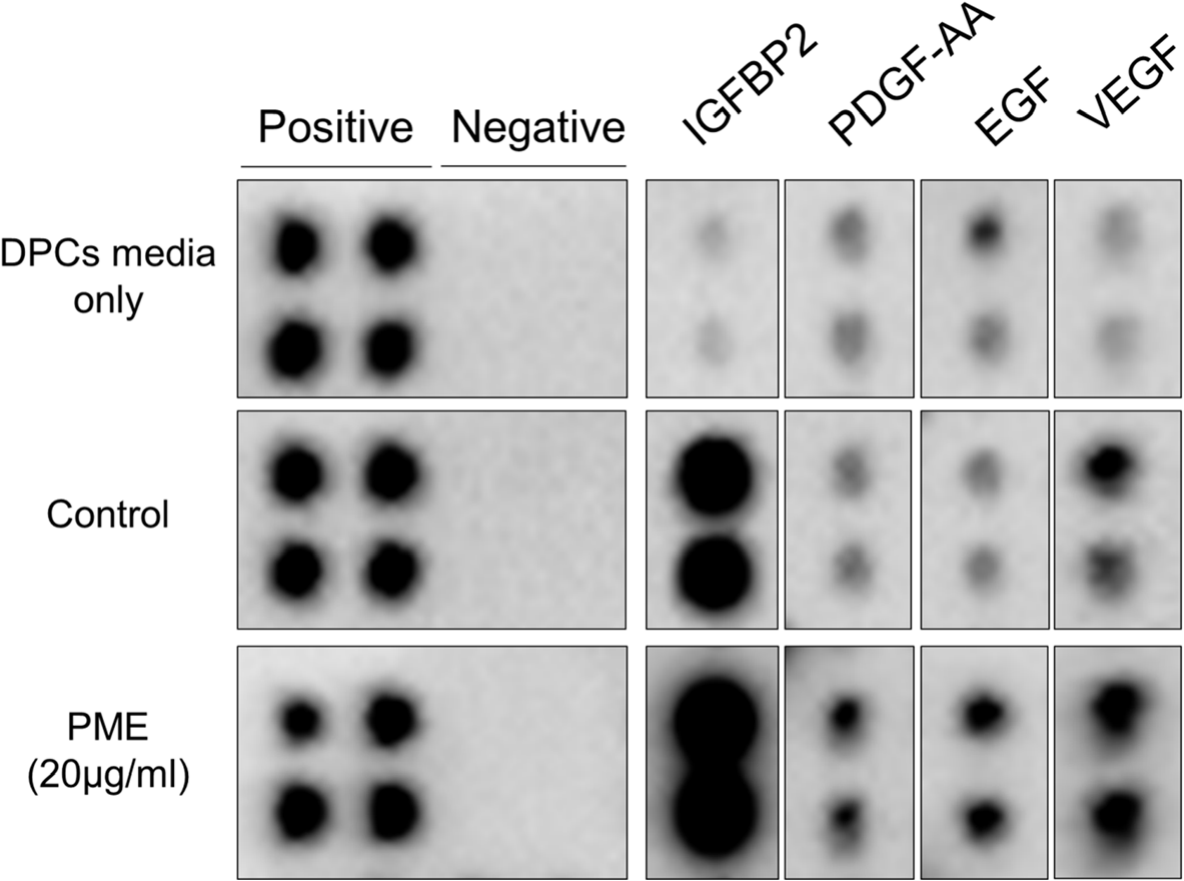
Effect of PM extract on growth factors secreted from DPCs. 41 types of growth factor analysis were performed with 20 μg/ml of PM extract treatment. PM extract treated DPCs’ supernatant was analyzed compared with DPCs growth media and culture supernatant of non-treated control

DPCs are known to interact with adjacent cells, such as hair germ cells, matrix progenitor cells, and outer root sheath cells. These interactions lead to hair follicle regeneration, hair shaft differentiation and hair cycle decision [30]. To investigate the changes in growth factors secreted in cultured DPCs by PM extract, growth factor analysis was performed using dot blot assay. The expression of IGFBP2, which controls the transcription of VEGF by regulating the binding of IGF family proteins to their receptors [31, 32], was markedly increased by PM extract (PM extract, 3rd row in Fig. 3). The expression of PDGF-AA, EGF, VEGF were also significantly increased by PM extract compared with control group (Fig. 3). IGFBP2, as mentioned above, regulates the binding affinity between IGF and IGFR by binding with IGF family. When existed as a singular form in extracellular matrix, IGFBP2 translocates to the cytoplasm and acts as a transcription factor for VEGF. VEGF is known to play an important role in mediating angiogenesis during hair growth cycle [33]. Stimulation of VEGF production by DPCs could explain the hair growth promoting potential of herbal extracts [34]. EGF is known to play an important role in anagen elongation or telogen escape, especially for telogen to anagen transition by stimulating EGFR located in the outer root sheath [35]. PDGF-AA also induces and maintains anagen of hair follicles. Studies have shown that injection of anti-PDGF antibody to mouse skin immediately triggered catagen and resulted in hair loss [36]. Our data strongly suggest that PM extract could support hair growth by stimulating the expression of growth factors essential for anagen induction and maintenance, e.g. IGFBP2, VEGF, EGF, and PDGF-AA.

### PM extract elongated anagen in human hair follicle organ culture

**Fig. 4:**
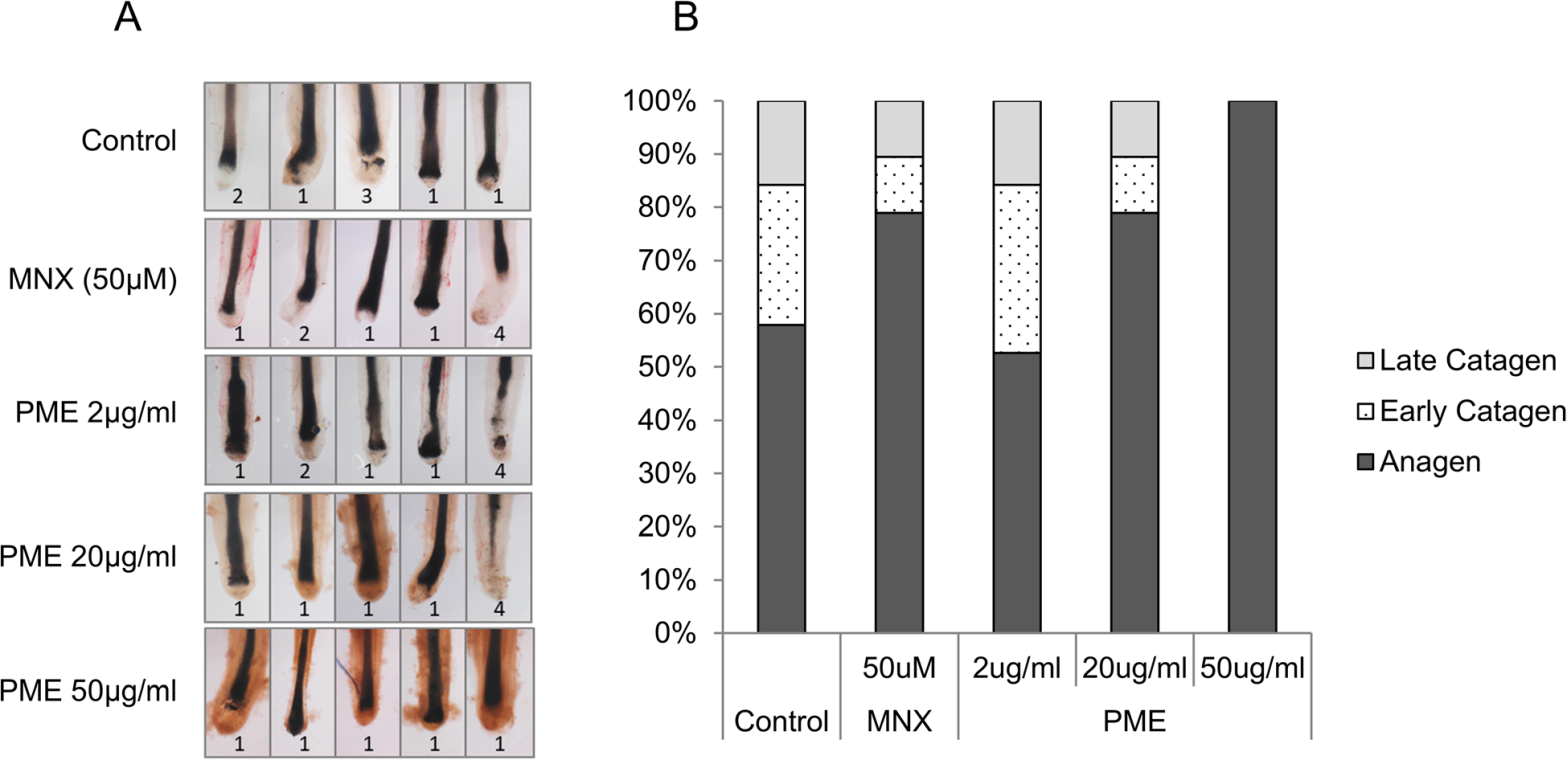
Effect of PM extract on anagen elongation and catagen entry in human hair follicle organ culture model. Human hair follicles (20 hair follicles/group) were treated with 2 μg/ml, 20 μg/ml and 50 μg/ml of PM extract and 50 μM of minoxidil. After 6 days of incubation, hair follicle morphology was assessed following hair cycle scoring criteria. **a** Representative images of hair follicles for each experimental group. **b** Calculated ratio of anagen, early categen, late catagen

As mentioned previously, PM extract could possibly elongate anagen by modulating the expression of several markers in cultured human DPCs, e.g. DKK-1, Bcl2, BAD, IGFBP2, EGF, VEGF and PDGF. To verify the effects of PM extract on human hair cycle, human hair follicle (hHF) organ culture model was adopted. During 6-day incubation period, 57.8% of hHF showed anagen hair follicle morphology in non-treated control group. In PM extract treated groups, on the other hand, the number of hair follicles in anagen was increased in a dose dependent manner. As shown in Fig. 4, treatment of PM extract at concentrations of 20 μg/ml and 50 μg/ml increased the ratio of hair follicles in anagen morphology to 78.9, 100%, respectively. Minoxidil, on the other hand, showed a comparable result with 20 μg/ml of PM extract treated group (Fig. 4a, b). Our data clearly demonstrates that PM extract could promote hair growth by extending anagen and delaying catagen entry in human hair follicles.

### Anti-androgenic effects of PM extract in cultured human DPCs

**Fig. 5:**
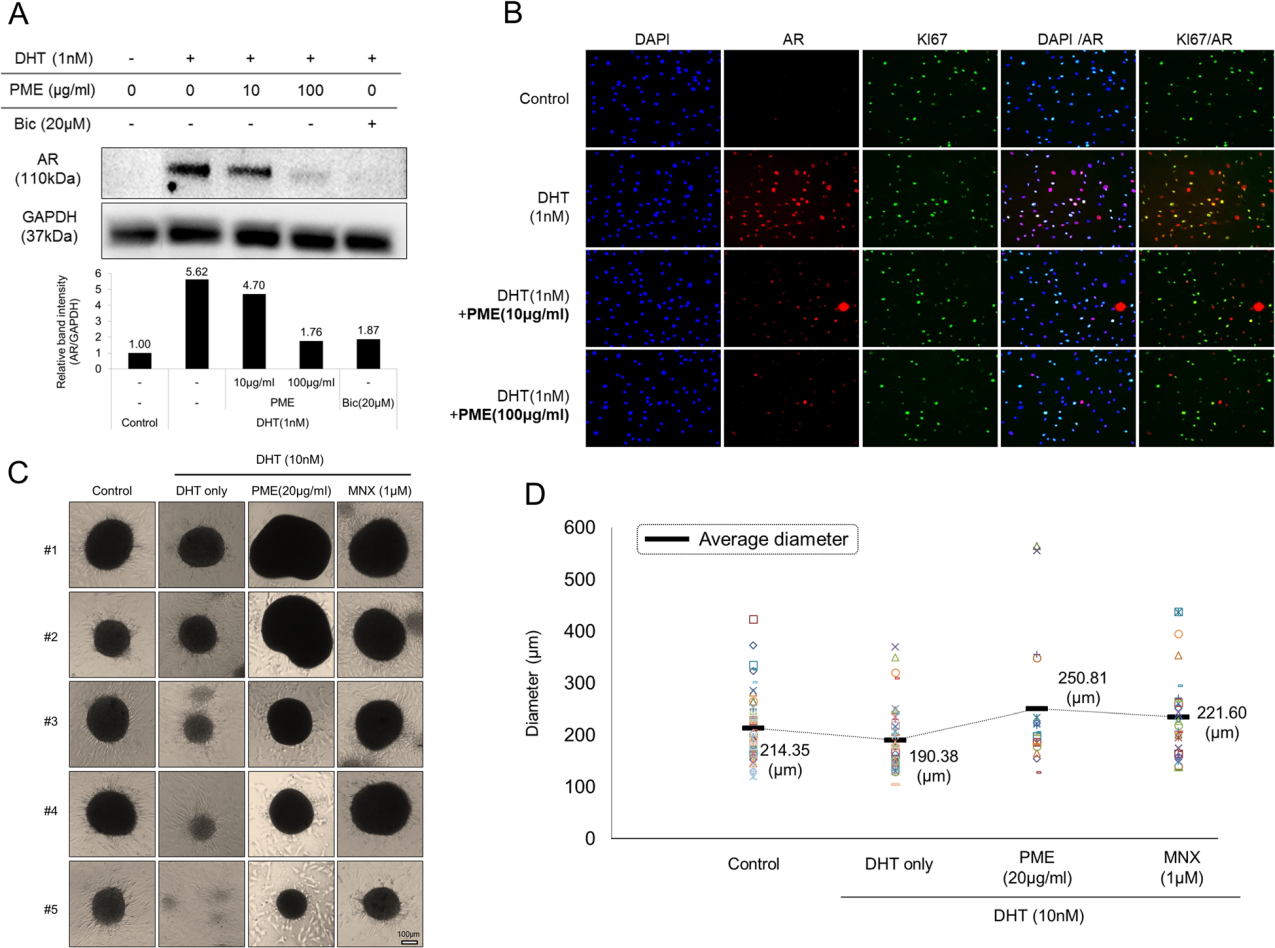
Anti-androgenic effect of PM extract on both 2D cultured DPCs and 3D DP spheroids. 2D cultured DPCs were co-treated with 1 nM of DHT and 10 μg/ml or 100 μg/ml of PM extract. Then, AR protein expression was analyzed by western blot or immunocytochemistry. For spheroids cultures, 10 nM of DHT was co-treated with 20 μg/ml of PM extract from the start of 3D DP spheroid formation. **a** AR protein was decreased by PM extract in dose dependent manner. **b** Nuclear AR was decreased by PM extract. **c, d** The size of spheroids was reduced by DHT treatment and recovered by PM extract as shown in representative images and calculated size distribution

AGA is caused by hair follicle miniaturization resulted from repeated hair cycles with shortened anagen that over time produces shorter and thinner hair [37, 38]. In DPCs, testosterone could be converted to more potent androgen dihydrotestosterone (DHT) by 5α-reductase in cytoplasm. Transformed androgen, DHT, binds to androgen receptor (AR) and then stimulate the expression of androgenic proteins which cause AGA [39]. In this context, anti-androgenic effects of PM extract in prostate cancer cell lines, 22RV1 and LNCap were investigated. Treatment of PM extract in 22RV1 cells transiently expressing pGL4.36 [*luc2P*/MMTV/Hygro] reporter vector markedly decreased the DHT induced AR response by 40% compared with DHT only treated group (*data not shown*). Moreover, prostate specific antigen (PSA) ELISA in LNCap cells showed that DHT-induced PSA level was significantly decreased by PM extract (*data not shown*). To investigate the possible anti-androgenic role of PM extract in preventing hair loss, the androgenic phenotypes in cultured human DPCs were examined. In cultured human DPCs, treatment of 1 nM DHT increased AR protein level by 562% and this increment was abrogated by PM extract in a dose dependent manner (Fig. 5a). Immunocytochemistry also showed that AR expression in the nucleus of DPCs was induced by DHT but significantly reversed by PM extract (Fig. 5b), suggesting that the previous anti-androgenic results obtained from prostate cancer cell lines were exerted by regulation of AR gene expression. To confirm anti-androgenic effect of PM extract*,* 3D DPC spheroids were constructed in the presence of DHT with/without PM extract. As shown in Fig. 5c, treatment of DHT at 10 nM concentration resulted in formation of spheroid-like small cell aggregates mostly under 100 μm in diameter, in contrast to the control group without DHT. Treatment of 20 μg/ml of PM extract, however, fully abrogated the inhibitory effect of DHT on spheroid formation and recovered the size of spheroid (Fig. 5c). PM extract increased the mean diameter (μm) of spheroids by 17 and 31% compared with non-treated and DHT treated control, respectively (calculated based on Fig. 5d). Minoxidil also recovered the size of DHT affected spheroids but the mean diameter was far less than that of PM extract treated group (Fig. 5d). The spheroid forming ability and the size of spheroids formed by cultured hair follicle DPCs are generally regarded as the hair inductive capacity and hair thickness, respectively [40, 41]. Also, because the expression of AR in balding hair is much higher than that in non-balding hair [42], our study suggests that PM extract could prevent androgen induced hair loss by reducing AR expression and preventing hair follicle miniaturization mediated by DHT which are thought to be the main cause of AGA.

## Discussion

Hair follicles are developmental organs. After neonatal follicle development has progressed, each hair follicle enters a regular cycle called anagen, catagen and telogen, results in repeating follicle formations and regressions. During catagen of the cycle, most of the cell types in hair follicles undergo apoptotic progression caused by activation of apoptotic ligands or mitochondrial dysfunctions and lead follicles to be phagocytized while DP remains intact [43]. This perpetuating property of DP is conferred by anti-apoptotic molecules such as Bcl-2 expressed in DP. AGA, also referred to as male pattern hair loss, is characterized by thinning of hair resulted from hair follicle miniaturization, leading to gradual replacement of large pigmented hairs (terminal hairs) by barely visible, unpigmented hairs (vellus hairs) in genetically predisposed individuals [44, 45]. Androgen plays a crucial role in accelerating hair loss by shortening of hair cycle period resulted from premature catagen induction with reduced anagen duration [46, 47].

The present report demonstrates the hair growth promoting effects of PM extract, especially focused on human DPCs, a key regulator of the hair cycle. We have found that PM extract enhanced cell viability and mitochondrial membrane potential in cultured human DPCs (Fig. 1a, b, c). This upregulated mitochondrial potential means that DPCs get harnessed with opposing force to apoptotic regression. We have found that the expression of DKK-1, which promotes catagen entry, was significantly decreased both in protein and mRNA levels by PM extract (Fig. 2a, c). Treatment of DKK-1 is reported to induce apoptosis in cultured outer root sheath keratinocyte cells through changing the expression of anti-apoptotic and pro-apoptotic proteins like Bcl-2, Bad, Bax, etc. [4, 48]. It is expected that PM extract might prolong anagen by delaying catagen entry, and this idea was further supported by the fact that PM extract increased the expression of Bcl-2 and decreased the expression of BAD (Fig. 2b, d and e).

It is well-documented in many reports that some growth factors are essential for hair growth. In this study, treatment of PM extract in cultured human DPCs stimulated the production of IGFBP2, VEGF, PDGF, and EGF, suggesting a possible underlying mechanism of hair growth promoting activity of PM extract (Fig. 3). The VEGF and VEGFR are essential proteins that support proliferation of DPCs and angiogenesis around hair follicles during hair cycle [33, 49]. IGFBP2 is also of importance because the transcription level of VEGF family is controlled by binding affinity of IGFBP2 to its receptor [31, 32]. PDGF, which mainly supplied from adipocyte near DP, is also expressed in DP and affects the DP itself [50, 51]. In terms of maintaining the size of DP, PDGF-AA increases the DP size by modulating stem cells in hair follicle [52]. EGF is well known growth factor for maturation of hair follicles, especially promoting proliferation of DP via notch signaling pathway [53].

By integrating all the results obtained from in vitro experiments, we raised a hypothesis that PM extract could support hair growth by stimulating growth factors and especially by elongating anagen. To verify our hypothesis, we performed an ex-vivo organ culture experiment for evaluating hair growth. As a result, PM extract extended anagen and prevented catagen induction of hair follicles in human hair follicle organ culture model. Our results strongly demonstrate the possible potential of PM extract as a new therapeutic agent for treating hair loss.

Because androgen plays a pivotal role in the process of AGA, searching for topically applicable anti-androgenic materials could be a promising therapeutic regimen for androgenetic hair loss. Anti-androgenic therapies are commonly performed in various methods like blocking androgen receptor, reducing the production of androgen, inhibiting 5-alpha-reductase activity etc. Also, pathogenesis of androgenetic alopecia patients displayed miniaturization of DP size via constant exposure to androgens [44]. In this context, PM extract was found to abrogate the DHT- induced AR expression almost completely and phenotypically recover DHT- induced DP miniaturization by enhancing spheroid forming capacity in 3D cultured human DPCs. Our data strongly suggest an anti-androgenic therapeutic potential of PM extract, possibly exerted by blocking of androgen receptor expression.

Natural product extract (NPE) is generally composed of thousands of chemicals with various biological activities, and this is why PM extract showed pleiotropic effects on cultured human DPCs. Even though we could not completely exclude the possibility that one chemical component of PM extract exerted all the hair growth promoting effects we have found, it seems plausible that several chemicals worked separately and/or cooperatively and this question is open for further investigation.

Although several reports demonstrated hair growth promoting effects of PM extract, the experiments were performed in mice and mouse cell lines [14, 54].

In this report, we have found that treatment of PM extract to cultured human DPCs showed hair growth supporting activities by stimulating cell proliferation and mitochondrial activity, decreasing the gene expression of DKK-1, and modulating the expression of Bcl-2 and BAD. In addition, PM extract stimulated the secretion of growth factors essential for hair growth, prominently abrogating the DHT-induced stimulation of androgen receptor (AR) expression, and inhibiting the increment of DP 3D spheroids size by DHT treatment. We also have found that PM extract prolonged the anagen stage of hHF in the human hair follicle organ culture model, that supports the possible therapeutic potential of PM extract for anti-hair loss treatment.

## Conclusions

In conclusion, our data suggest that PM extract could promote hair growth possibly through prolongation of anagen by preventing catagen entry with increased production of growth factors and abrogating the effects of androgen, DHT.

## Supplementary information

**Additional file 1.**

**Additional file 2.**

## Data Availability

The datasets used and/or analyzed during the current study available from the corresponding author on reasonable request.

## Abbreviations

22RV1: The cell line expresses prostate specific antigen
BAD: Bcl2 associated agonist of cell death
Bcl2: B-cell lymphoma2
BSA: Bovine serum albumin
CCK8: Cell counting kit8
DAPI: 4′,6-diamidino-2-phenylindole
DHT: Dihydrotestosterone
DKK1: Dickkopf-related protein 1
EGF: Epidermal growth factor
EGFR: Epidermal growth factor receptor
ELISA: Enzyme-linked immunosorbent assay
HGF: Heptocyte growth factor
hHF: Human hair follicle
IGF1: Insulin-like growth factor1
IGFBP2: Insulin-like growth factor binding protein 2
JC1: Mitochondrial membrane potential kit
KGF: Keratinocyte growth factor
LNcap: The cells which are responsive to 5-alpha-dihydrotestosterone
MMTV: Mouse mammary tumor virus
NADH: Reduction form of nicotinamide adenine dinucleotide
NADPH: Reduction form of nicotinamide adenine dinucleotide phosphate
PDGF: Platelet-derived growth factor
PSA: Prostate specific antigen
TGF: Transforming growth factor
VEGF: Vascular endothelial growth factor
