# Supplementary material for: Polygonum multiflorum extract support hair growth by elongating anagen phase and abrogating the effect of androgen in cultured human dermal papilla cells
Source: BMC Complement Med Ther. 2020 May 12;20:144. doi: 10.1186/s12906-020-02940-5 (PMC7218528; doi:10.1186/s12906-020-02940-5)
Supplement: Supplementary file 1 — Additional file 1. [file 12906_2020_2940_MOESM1_ESM.docx]

**Supplementary Material :** HPLC analysis results of *polygonum multiflorum*(PM) extract

**Materials and Methods** : To identify constituents of *polygonum multiflorum*(PM) extract, HPLC analysis was performed. The dried roots of PM were extracted with 50% aqueous ethanol (1:20) for 2 days at room temperature, filtered through Whatman No. 4 filter paper. The filtrate was concentrated by rotary evaporator under reduced pressure to give 50% aqueous ethanol extract. The extract was diluted 200 fold in 50% methanol. For quantitation, an external standard method was utilized. Gallic acid, catechin, epicatechin, 2,3,5,4-Tetrahydroxystilbene 2-O-β-D-glucoside (TSG) and emodin were used as standards of *polygonum multiflorum*(PM) extract as previously reported. The stock solutions of each standard were prepared in 50% methanol just before use. The standards were purchased from Sigma (St. Louis, MO, USA, gallic acid, catechin, epicatechin, emodin) and USP (USA, TSG). The analytical conditions were described in Table S1.

**Table S1**. Analytical conditions of HPLC analysis

| HPLC test method | Time  (min) | Flow rate  (ml/min) | 0.05% H3PO4 (%) | ACN (%) |
| --- | --- | --- | --- | --- |
| - Agilent 1260 Infinity II system  -Column: zorbax SB-AQ column  (50mm × 4.6mm, 3.5μm)  -Column temperature: 30℃  -Injection volume: 10 μl  -Detector: HPLC-DAD  (210, 280 nm)  -Eluent | 0 | 1.0 | 100 | 0 |
|  | 7 | 1.0 | 94 | 6 |
|  | 12 | 1.0 | 94 | 6 |
|  | 20 | 1.0 | 92 | 8 |
|  | 22 | 1.0 | 88 | 12 |
|  | 50 | 1.0 | 75 | 25 |
|  | 55 | 1.0 | 10 | 90 |
|  | 65 | 1.0 | 10 | 90 |
|  | 66 | 1.0 | 100 | 0 |
|  | 72 | 1.0 | 100 | 0 |

Table S2. Contents of Phenolic Compounds in the *polygonum multiflorum* Extract

| No. | Phenolic compound | Content (μg/mL) | t_R_ (min) |
| --- | --- | --- | --- |
| 1 | Gallic acid | 680 | 3.1 |
| 2 | Catechin | 320 | 11.2 |
| 3 | Epicatechin | 200 | 16.4 |
| 4 | TSG | 62,000 | 28.8 |
| 5 | Emodin | 3,200 | 54.0 |

**RESULTS** : Fig. S1 shows the HPLC profiles of the phenolic compound standards (A, C) and phenolic compounds in PM Extract (B, D) at 210nm and 280nm. The contents and retention times of the phenolic compounds were showed in Table S2.

A
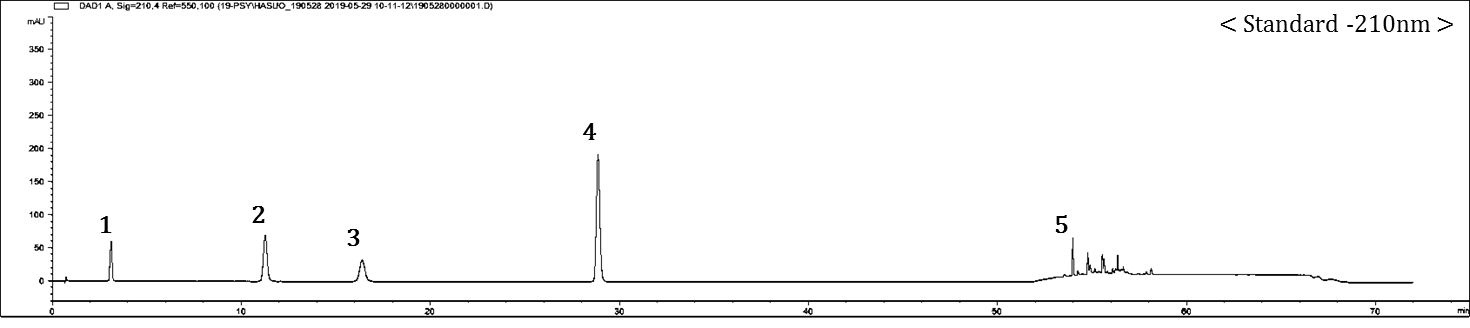


B
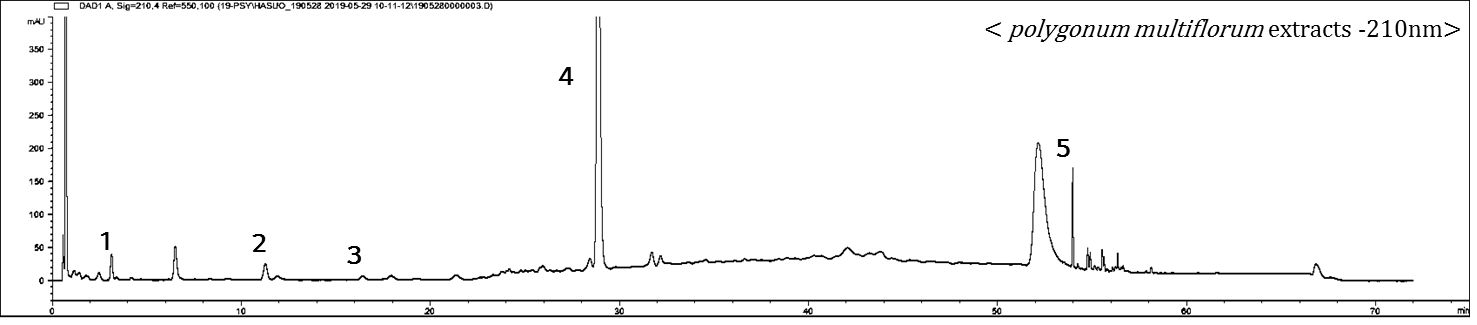


C
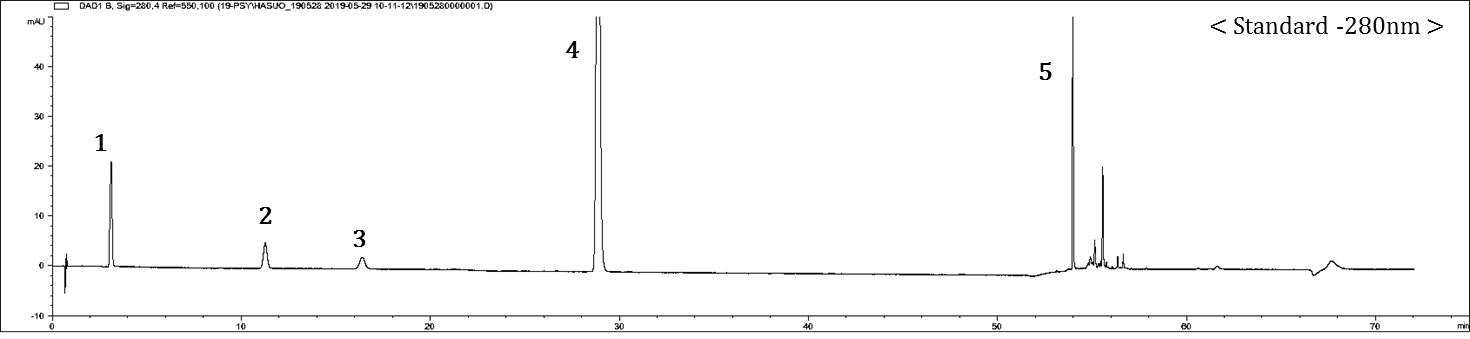


D
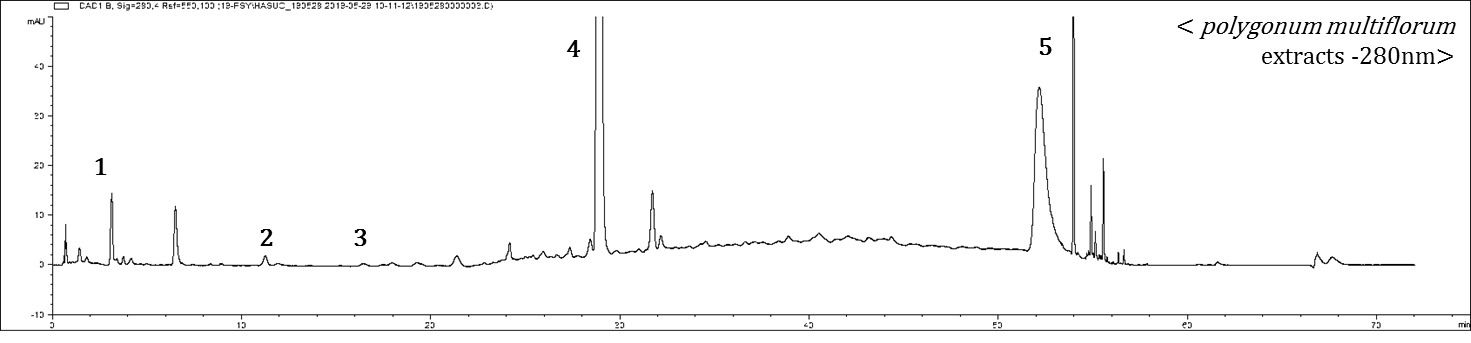


**Fig. S1**. HPLC profiles of the phenolic compound standards **(A, C)** and phenolic compounds in PM Extract **(B, D)** at 210nm and 280nm, respectively. The PM extract was diluted 200 fold in 50% methanol. Peaks : (1) gallic acid, (2) catechin, (3) epicatechin, (4) 2,3,5,4’-tetrahydroxystilbene 2-O-β-D-glucoside (TSG), and (5) emodin .

**REFERENCES**: Han D.Q., Zhao J., Xu J., Peng H.S., Chen X.J., & Li S.P. (2013) Quality evaluation of Polygonum multiflorum in China based on HPLC analysis of hydrophilic bioactive compounds and chemometrics. *Journal of Pharmaceutical and Biomedical analysis*, **72**, 223-230
