# Supplementary material for: Polygonum multiflorum extract support hair growth by elongating anagen phase and abrogating the effect of androgen in cultured human dermal papilla cells
Source: BMC Complement Med Ther. 2020 May 12;20:144. doi: 10.1186/s12906-020-02940-5 (PMC7218528; doi:10.1186/s12906-020-02940-5)
Supplement: Supplementary file 2 [file 12906_2020_2940_MOESM2_ESM.docx]

Target gene		Target CT	GAPDH CT	delta CT (dCT)	Average dCT (Control)	delta dCT (ddCT)	2^-ddCT	Average 2^-ddCT	Relative mRNA expression	STD	*p* -value (Control)
BCL2	Control	35.38	22.65	12.73	12.713333	0.01667	0.98851	1.00004	1.0000	0.01057	
		35.27	22.57	12.70		-0.01333	1.00928				
		35.32	22.61	12.71		-0.00333	1.00231				
	PME 20μg/ml	35.56	23.09	12.47		-0.24333	1.18372	1.24962	1.2496	0.06968	* 0.02556
		35.4	23.09	12.31		-0.40333	1.32256				
		35.48	23.08	12.40		-0.31333	1.24258				
	PME 50μg/ml	35.38	23.18	12.20		-0.51333	1.42734	1.50283	1.5028	0.06877	* * 0.00632
		35.25	23.18	12.07		-0.64333	1.56193				
		35.31	23.2	12.11		-0.60333	1.51922				
BAD	Control	33.01	22.65	10.36	10.340000	0.02000	0.98623	1.00005	1.0000	0.01196	
		32.9	22.57	10.33		-0.01000	1.00696				
		32.96	22.63	10.33		-0.01000	1.00696				
	PME 20μg/ml	34.03	22.71	11.32		0.98000	0.50698	0.54550	0.5455	0.03772	** 0.00252
		33.79	22.67	11.12		0.78000	0.58237				
		33.91	22.7	11.21		0.87000	0.54715				
	PME 50μg/ml	33.86	23.18	10.68		0.34000	0.79004	0.73190	0.7319	0.05821	* 0.01598
		34.09	23.18	10.91		0.57000	0.67362				
		33.99	23.2	10.79		0.45000	0.73204				
